# Web-Based Development of Standard Operating Procedures and Midwifery Trainings at Ugandan Birth Clinic in the Framework of Implementing a Quality Improvement System for the MEWU—Midwife Exchange with Uganda

**DOI:** 10.1089/whr.2024.0068

**Published:** 2024-10-07

**Authors:** Lea Stubbe, Anja Philippeit, Jill Philippeit, Laura Kaukemüller, Markus Kruppa, Marie Sunder-Plassmann, Alicia Ruppert, Peter Hillemanns, Jerome Mugisha, Rüdiger Klapdor

**Affiliations:** ^1^ Department of Gynecology and Obstetrics, Hannover Medical School, Hannover, Germany.; ^2^ Department of Gynecology, KRH Clinic Siloah, Hannover, Germany.; ³Medical care center Winterhude, Hamburg, Germany.; ^4^ Department of Obstetrics, St. Francis Hospital Mutolere, Mutolere, Uganda.

**Keywords:** midwifery, internet-based intervention, education, global health, maternal health

## Abstract

**Introduction:**

High maternal and newborn mortality rates in Sub-Saharan Africa indicate the need for global action interventions. Thus, the clinic cooperation midwife exchange with Uganda (MEWU) between Hannover Medical School and Mutolere Hospital, Uganda, was founded. This study, as the first intervention within the MEWU framework, explored whether a web-based approach is suitable for developing, training, and establishing standard operating procedures (SOPs) at Mutolere Hospital. We focused on assessing midwives’ confidence in midwifery core competencies.

**Methods:**

The study was conducted in a prospective, non-controlled intervention design. As a quality improvement tool, the Plan, Do, Check, Act cycle was implemented. SOPs for standard obstetric care were developed and trained in online meetings. Qualitative and quantitative data were collected through a questionnaire completed pre- and post-intervention by participating midwives, evaluations, minutes and video recordings of each case training, and annual analytical reports from Mutolere Hospital containing morbidity and mortality data.

**Results:**

The online intervention was successfully implemented. Nine SOPs on basic obstetric care were developed, trained in online case training, and integrated into clinical practice at Mutolere Hospital. An increase in midwives’ confidence regarding all surveyed core competencies was observed. There were no significant changes in the hospital’s morbidity and mortality rates. The quality management system was implemented to optimize the follow-up projects further.

**Conclusion:**

This pilot study shows the potential of web-based interventions as a quality improvement tool in developing countries. The developed SOPs and video database are being used in subsequent studies and extended to further health centers in the Kisoro region.

## Introduction 

Every day approximately 830 women die from pregnancy-related complications around the world. The global maternal mortality ratio (MMR) is still high at 223 per 100.000 live births.^1^The East African country Uganda continues to have an exceedingly high MMR of 284 per 100,000 live births in 2020^2^ despite significant improvement during the last decades.

Furthermore, Uganda also has a high neonatal mortality rate (NMR) of 27 deaths per 1000 live births.^3^ The majority of neonatal deaths (28.6%) occur due to asphyxia and trauma during birth.^4^ The UN’s sustainable development goals call for eliminating preventable deaths, reducing global maternal mortality to less than 70 per 100,000 live births, and lowering neonatal mortality to 12 or fewer deaths per 1,000 live births by 2030.^5^

A great challenge in Uganda’s health system is the shortage of skilled birth attendants and limited mentorship, training, and supervision of health workers.^6–9^ The private nonprofit clinic Mutolere is involved in meeting this challenge and investing in its affiliated midwifery training school. The birth clinic Mutolere serves as a referral clinic for many health centers in the Kisoro region. Yet, clinical staff is limited, with 19 midwives and two obstetricians responsible for delivering around 1500 babies per year, of which on average more than 500 are born *via* caesarean section.^10^ In 2020, the midwife exchange with Uganda (MEWU) was founded between Hannover Medical School (MHH) and Mutolere Hospital. The idea was to visit the partner hospitals, to learn from one another and to improve the midwifery work.

This study focuses on establishing standard operating procedures (SOPs) to standardize processes in the labor ward. In the context of evidence-based medicine, SOPs are internationally established guidelines.^11^ Research shows that the implementation of SOPs has favorable effects on patient outcomes.^12,13^

Zamboni’s systematic review from 2020 suggests, that quality improvement collaboratives in low- and middle-income countries (LMICs), such as Uganda, can achieve numerous positive outcomes.^14^ According to the recent Lancet global health study (2020) in Uganda and Kenya, the use of quality management interventions combined with midwifery training was successful in reducing the rate of fresh stillbirths significantly.^15^

The aim of this study was to implement a quality management system to improve midwifery care at Mutolere Hospital. We evaluated the feasibility of implementing a web-based midwife exchange and explored whether this approach is suitable for developing and establishing SOPs at Mutolere Hospital. Furthermore, we assessed a potential change of confidence among midwives in their own knowledge and professional skills. Secondarily, we evaluated the change in morbidity and mortality rates during the study period.

## Methods

### Study design and population

The study was conducted in a prospective, non-controlled intervention design. The study period was set between July 2020 to June 2021. Participants were 31 Ugandan midwives and midwifery students as well as four tutors from Mutolere Hospital’s midwifery school.

### Stages of study—Plan, Do, Check, Act cycle

**FIG. 1.**
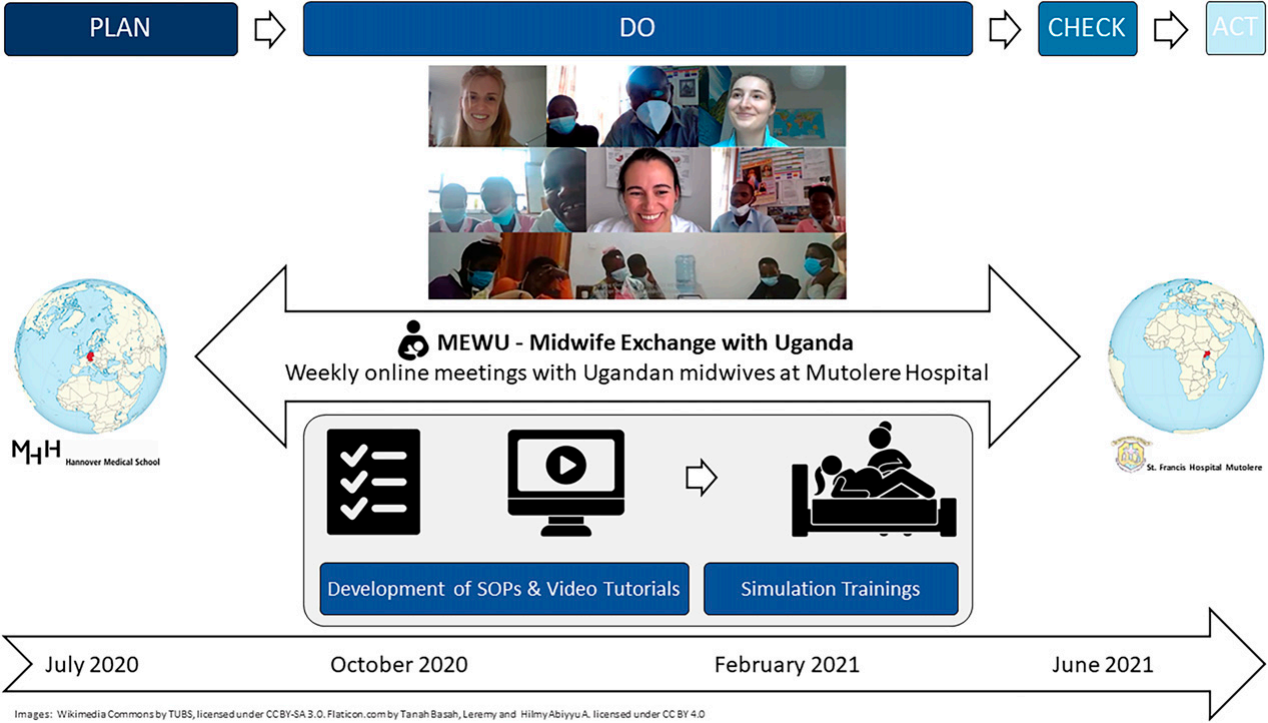
Study concept.

The study was structured according to the Plan, Do, Check, Act (PDCA) cycle, which is divided into the four phases plan, do, check, and act (Fig. 1).

Plan: standard operating procedures (SOPs) topics and time schedules for the exchange were developed in cooperation with the Ugandan project partners. Equipment for the video exchange was established.

Do: the exchange was conducted in an online format *via* the platform zoom on a weekly to fortnightly basis.

Check: study effects were measured at two specific reference points pre- and post-intervention. Additionally, each online meeting was assessed through pertinent evaluation tools (see below).

Act: based on study findings, adjustments were made for subsequent years of the MEWU. In this way, the exchange is constantly optimized in terms of quality improvement.

### Evaluation tools

- A pilot questionnaire based on the nurse practitioner primary care organizational climate questionnaire (NP-PCOCQ)^16^ was developed. The questionnaire consisted of 32 statements regarding midwives’ confidence in their level of knowledge and skills, such as “I feel confident monitoring the fetal heart rate”, and regarding the overall practice climate. Participants could assess to what extent they agreed with each item. We adopted the ordinal Likert rating scale from the NP-PCOCQ. Thus, agreement values can be translated into numerical values on a scale from 1 “I strongly disagree” to 4 “I strongly agree.” The questionnaire was filled in pre-intervention before the first online meeting in October 2020 and post-intervention in July 2021, after the last online meeting of this study was conducted.
- Detailed minutes of all online meetings recorded discourse and midwives’ participation. Video recordings of the online case training enabled precise analysis of setting data.
- An additional evaluation form was specifically tailored to the case trainings. It allowed the midwives to rate the design of each training as well as its effects on their confidence and to assess the acceptance of the respective SOP by the midwives, using the ordinal Likert scale. Blank text fields provided space for free feedback.
- In the form of annual analytical reports (AAR) morbidity and mortality data from Mutolere Hospital were collected (from July 1, 2020, to June 30 of the following year). The data encompass the rates for caesarean section, infected caesarean section wounds, maternal mortality, stillbirths, and early neonatal deaths.

### Statistical analysis

We used GraphPad Prism^©^ (Version 9.2.0) to conduct our analyses. We used the chi-square test for categorical data and the Mann–Whitney U-test for continuous data. A *p*-value below 0.05 was considered significant.

### Ethical considerations

The study protocol conforms to the ethical guidelines of the Declaration of Helsinki.^17^ Informed consent was obtained from all participants before conducting this study. Participation in the study was voluntary. The data records of all surveys are available in anonymized form. The ethics committee of Hannover Medical School approved this study *a priori* (Nr. 9330_BO_K_2020).

## Results

### The video exchange was carried out successfully over 22 meetings

The online meetings took place between October 2020 and June 2021 on a weekly to fortnightly basis. With an average of 12 (5–15) participants, the outreach was consistently very high. Minutes of the meetings recorded a total of 34 midwives and midwifery students taking part in the exchange. Video recordings of the case training show a mean duration of 58 (45–69) minutes, with a mean interruption due to a poor internet connection of 5 (0–11) minutes.

### Nine SOPs on basic obstetric care were developed in online meetings with the Ugandan midwives

The SOPs were based on WHO guidelines and specifically adapted to the conditions at Mutolere Hospital in close collaboration with the Ugandan midwives. Content-wise, the SOPs cover core midwifery competencies, namely patient admission, partogram, fetal heart rate, spontaneous delivery, caesarean section, operative-vaginal delivery, newborn care, newborn examination and immediate newborn care.

**FIG. 2.**
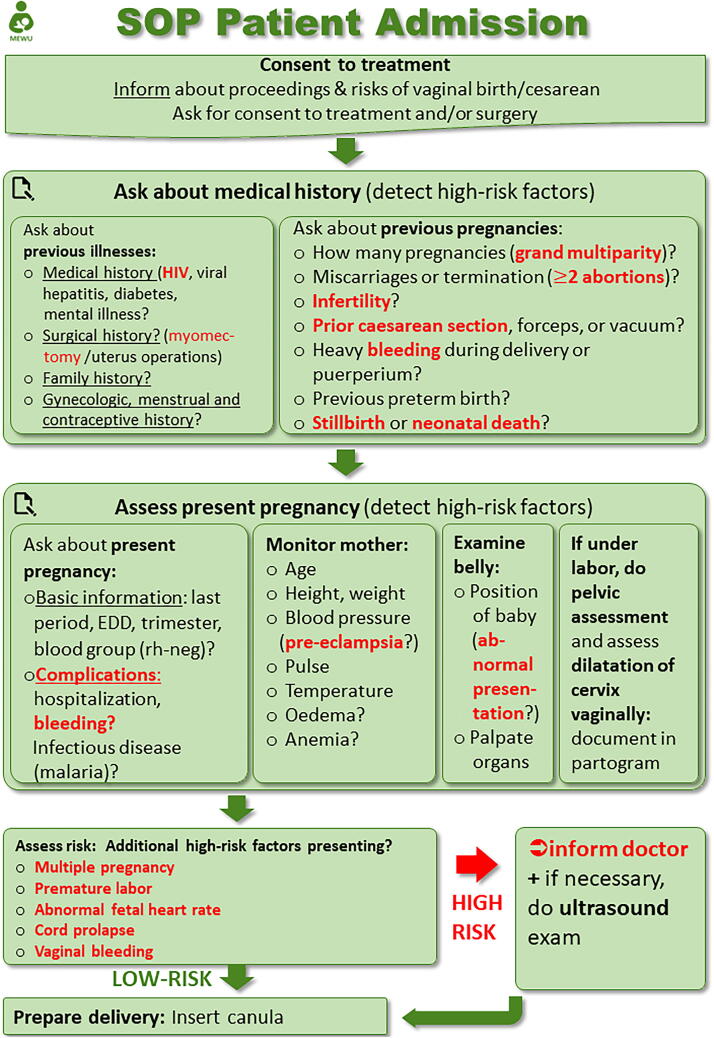
SOP patient admission. SOP, standard operating procedure.

The SOPs consist of step-by-step practical guidelines for midwives, highlighting so-called “red flags,” that require immediate medical attention. Our study group created concise flowcharts for each developed SOP (Fig. 2) for the midwives to keep in the form of a printed copy.

In a second sequence of online meetings between February and May 2021, the contents of each SOP were consolidated in so-called “case training” by means of practical examples. Our project group created staged cases, which allowed us to treat model patients following the instructions of the respective SOP. The project group of MHH as well as the Ugandan midwives participated in simulations. To increase the learning effect, video tutorials that take the midwives through the respective SOP step by step were created and shown at the end of each case training.

### The questionnaire registered a clear shift toward a higher level of confidence among midwives post-intervention

The midwives’ mean confidence score regarding all areas surveyed in the questionnaire increased significantly by 0.44 score points, from pre-interventional 3.19 to post-interventional 3.63 score points (*p* < 0.01). Firstly, the results of the questionnaire show an increase in midwives’ confidence in their own knowledge and skills. This not only includes everyday obstetric care, for example, filling out the partogram, monitoring the fetal heart rate, or guiding a spontaneous delivery, but also more advanced obstetric care, such as detecting maternal complications postpartum or assisting operative-vaginal delivery (OVD).

**FIG. 3.**
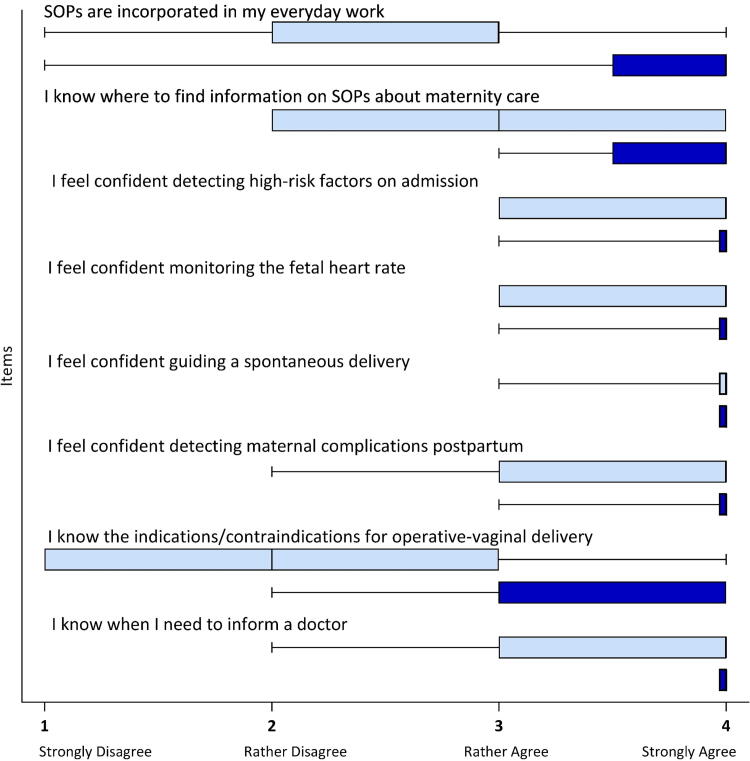
Integration of SOPs into clinical practice and midwives’ confidence pre- and post-intervention.

Furthermore, we identified a tendency toward higher levels of midwives’ confidence in differentiating high-risk from low-risk patients. This topic was particularly emphasized in the SOPs. The greatest increase in confidence was registered in the field of OVD (Fig. 3). Here the confidence of midwives increased by up to 1.2 score points [0–3].

The questionnaire item “I know when I need to inform a doctor” deserves special mention. This statement was unanimously agreed upon by the 16 evaluating midwives post-intervention, resulting in a maximum score of 4.0 (Fig. 3).

In addition, a post-interventional trend toward a more positive workplace culture and a better relationship between doctors and midwives was identified. A significant mean increase of 0.85 score points (*p* < 0.01) from a mean score of 2.6 pre-intervention to 3.45 post-intervention was registered in the questionnaire items regarding teamwork. The largest increase of a mean of 1.2 score points was observed with regard to the questionnaire items “I regularly get feedback about my performance from my practice leadership” and “Practice leadership is open to midwives’ ideas to improve patient care.”

Moreover, the questionnaire indicates high acceptance of the video exchange by the participating midwives and the successful implementation of SOPs into clinical practice at the maternity ward. The latter is highlighted by the fact that midwives’ agreement with the item “SOPs are incorporated in my everyday work” increased from 2.7 [1–4] score points pre-intervention to 3.6 [1–4] score points post-intervention (Fig. 3).

### Evaluation form findings showed that the case trainings were rated as an overall positive intervention and succeeded in increasing midwives’ confidence

The evaluation forms were filled in after each case training and allowed for more detailed evaluation as they concern only one SOP at a time. In accordance with the results of the questionnaire, the item “it is feasible to integrate the SOP into my work routine” was rated with a mean score of 3.8 [3–4] by the participating midwives. The midwives rated the case training as a positive intervention that clarified the clinical meaning of the SOPs, with an overall approval score of 3.8 out of 4 (“The case training helped me to understand the clinical meaning of the SOP”).

Furthermore, the case training affected midwives’ confidence positively. Thus, the midwives rated the statement “The case training gave me confidence in my work routine” with a mean agreement score of 3.9 out of 4.0.

### Changes in morbidity and mortality were statistically not significant

**FIG. 4.**
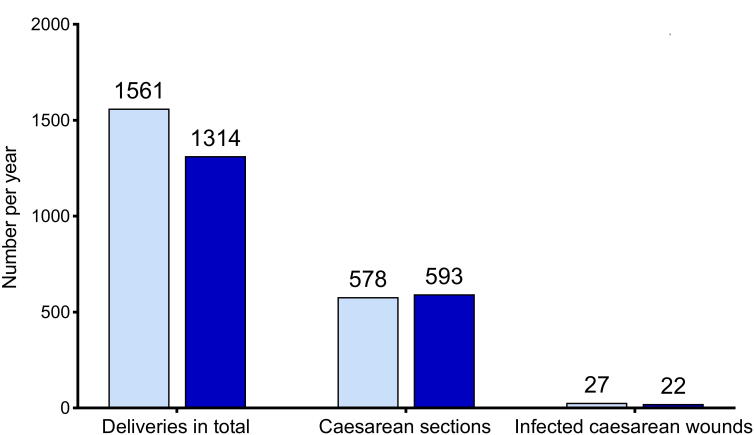
Morbidity data from Mutolere Hospital pre- and post-intervention.

**FIG. 5.**
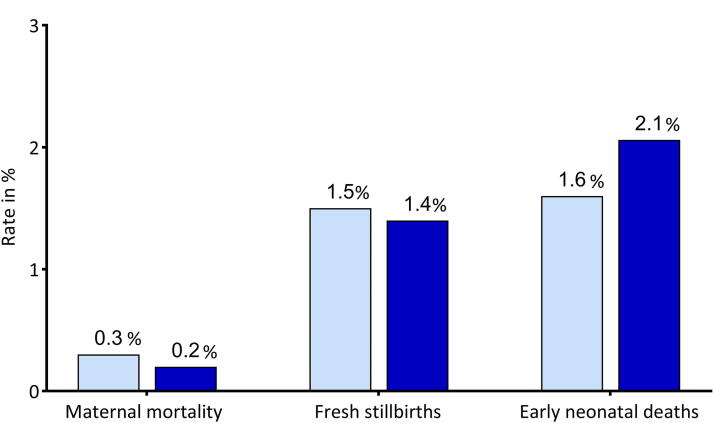
Mortality data from Mutolere Hospital pre- and post-intervention.

The AAR of Mutolere Hospital show a small reduction in morbidity post-intervention (post-operative infection rate after caesarean section) (Fig. 4). However, the caesarean section rate overall has risen strongly. The maternal mortality rate and the rate of stillbirths were reduced by 0.1% each (*p* = 0,72), in the year of the intervention. The early NMR increased by 0.5% (*p* = 0,43) over the same period (Fig. 5). A causal relation with the intervention is not evident.

## Discussion

### Summary

Our hypothesis, that it is feasible to conduct a midwife exchange in an online format, and that this approach would be successful in developing and establishing SOPs on basic obstetric care at Mutolere Hospital, was confirmed.

Our intervention was effective in equipping the clinic for the video exchange. Thus, 22 online meetings were successfully conducted, with a mean outreach of 12 midwives per meeting. Nine SOPs were developed, trained in online case training, and integrated into clinical practice at Mutolere. A questionnaire filled out pre- and post-intervention by the participating midwives showed an increase in confidence regarding all surveyed core competencies of midwifery work.

### Study design in an online format

We took an online approach to our intervention for its timeliness and research relevance. Scientific literature shows that web-based medical training is generally an effective tool to increase expertise and enhance participants’ confidence in the trained area.^18–21^ However, web-based training explicitly for midwives has not been sufficiently explored.^22^ Yet, recently several Ugandan midwifery institutions have started E-learning programs.^23,24^ This highlights the relevance of online approaches in this field. Clear benefits of the online approach in our exchange with Mutolere Hospital are its cost-effectiveness in a resource-limited setting, its potential to easily expand the project to include more midwives and health centers, as well as its sustainability in maintaining the exchange in the long run. According to UNICEF estimates, the COVID-19 pandemic has halted the immense progress that had been achieved in maternal and perinatal health in the past decades, with maternal mortality increasing again in 2020.^25,26^ In light of the pandemic, the prospect of contactless exchange is of particular relevance.

### Study findings in the context of research literature

Because peer-reviewed studies on the effectiveness of in-service midwifery education in LMICs are limited,^27^ we believe our study makes a valuable contribution to this field of research. We have placed the focus of our study on midwives, due to their great potential to positively impact maternal and newborn health. The Lancet’s modeling study estimates that universal coverage of midwife-delivered interventions could prevent 67% of maternal deaths, 65% of stillbirths, and 64% of neonatal deaths.^28^ However, according to the state of the world’s midwifery report of 2021, further investment in education and training is needed for midwives to reach their full potential.^29^ Therefore, interventions centered around midwifery training are crucial.

Midwifery simulation- and skills training have already proven to be a pertinent measure to increase midwives’ confidence and close the knowledge-to-practice gap.^30–32^ Our study results indicate to be consistent with those research findings, as the item” The case training gave me confidence in my work routine” was evaluated with an overall score of 3.9 out of 4.0 [3.5–4], and the item “it is feasible to integrate the SOP into my work routine” achieved a mean confidence score of 3.8 [3,6–4] post-intervention.

Our study showed a post-interventional increase in midwives’ confidence concerning all surveyed core competencies. This represents a major success as evidence demonstrates that strengthening practitioner’s confidence leads to improved clinical decision making^33,34^ and confidence is a fundamental component of clinical practice and a key determinant of clinical ability and competence.^35,36^

Retention of trained skills is temporary.^37^ Research suggests, that simulation-based, low-dose, high-frequency training may help maintain and even continue to improve health workers’ knowledge and competencies.^38,39^ Therefore, to ensure the lasting effects of our intervention we put follow-up studies with continuous training into place. It will be interesting to look at the forthcoming data and assess potential changes in midwives’ confidence in their own knowledge and skills over the course of multi-year continuous training.

Other midwife exchanges, such as the momentum project in Uganda,^40^ have been successful in creating an enabling community of practice. This result could be replicated in our study, as our analysis showed a positive development toward a more favorable community of practice at Mutolere Hospital. An enabling work environment is a prerequisite for midwives to realize their full potential^41^ and to contribute to the reduction of maternal and perinatal mortality.^28,41,42^

### Limitations

This pilot study was limited in size and scope. The small study population of 36 participants limited the power of the study. Due to the study design as a single-arm intervention study, the study results cannot be juxtaposed with a control group. The implementation of the PDCA cycle proved to be a suitable method for continuous quality improvement at Mutolere Hospital. However, it should be regarded as critical that the act-phase of the PDCA cycle is not applied in this study but appears only in the form of adaptations for follow-up projects.

As a feasibility study, no changes in long-term outcomes such as morbidity and mortality were expected. In addition, the COVID-19 pandemic represents a significant confounder for the evaluation of these secondary endpoints. Morbidity and mortality will be important endpoints in future follow-up studies.

## Conclusion

This pilot study demonstrates the feasibility of implementing an online midwife exchange in a Ugandan birth clinic. Our hypothesis, that a web-based intervention can be successful in developing and training SOPs on basic obstetric care at Mutolere Hospital proved to be true. The intervention was successfully conducted, resulting in a significant increase in midwives’ confidence. The online format proved advantageous in terms of cost-effectiveness, sustainability, and scalability. Hence, the developed SOPs and video database were scaled up to reach other health centers in the Kisoro region, ensuring a long-term impact of the intervention.

## Abbreviations Used

AAR: Annual Analytical Report
LMIC: Low- and Middle-Income Country
MEWU: Midwife Exchange with Uganda
MHH: Hannover Medical School
MMR: Maternal Mortality Ratio
NMR: Neonatal Mortality Rate
NP-PCOCQ: Nurse Practitioner Primary Care Organizational Climate Questionnaire
OVD: Operative Vaginal Delivery
PDCA: Cycle Plan-Do-Check-Act Cycle
QM: Quality Management
QMS: Quality Management System
SDG: Sustainable Development Goal
SOP: Standard Operating Procedure
UN: United Nations
UNICEF: United Nations International Children’s Emergency Fund
WHO: World Health Organization
